# Pancreatic cancer microenvironment: a current dilemma

**DOI:** 10.1186/s40169-019-0221-1

**Published:** 2019-01-15

**Authors:** Burak Uzunparmak, Ibrahim Halil Sahin

**Affiliations:** 1 The University of Texas MD Anderson Cancer Center, Houston, USA; 2 Winship Cancer Institute, Emory University School of Medicine, Atlanta, USA

**Keywords:** Pancreatic cancer, Microenvironment, Immunotherapy, Targeted therapy, Desmoplasia, Sonic hedgehog, Sonic hedgehog inhibitors, Chemosensitization, Hyaluronan

## Abstract

Pancreatic cancer is one of the leading causes of cancer-related death in the United States and survival outcomes remain dismal despite significant advances in molecular diagnostics and therapeutics in clinical practice. The microenvironment of pancreatic cancer carries unique features with increased desmoplastic reaction and is infiltrated by regulatory T cells and myeloid-derived suppressor cells which negatively impact the effector immune cells. Current evidence suggests that stellate cell-induced hypovascular stroma may have direct effects on aggressive behavior of pancreatic cancer. Preclinical studies suggested improvement in drug delivery to cancer cells with stroma modifying agents. However these findings so far have not been confirmed in clinical trials. In this article, we elaborate current-state-of-the science of the pancreatic cancer microenvironment and its impact on molecular behavior of cancer cells, chemotherapy resistance and druggability of stroma elements in combination with other agents to enhance the efficacy of therapeutic approaches.

## Introduction

Pancreatic cancer is one of the most challenging cancers among gastrointestinal malignancies due to various factors including aggressive molecular behavior driven by the loss of multiple tumor suppressor genes [1, 2], lack of effective immune response with low immunogenicity [3] and complex tumor microenvironment [4]. To date, the mainstay treatment of pancreatic cancer is a combination of cytotoxic agents in adjuvant and metastatic settings [5, 6]. Despite efforts in the past decades, targeted therapy approaches have not yielded substantial improvement in clinical outcomes essentially due to complex signaling pathways [7]. Immunotherapeutic agents have also been investigated in pancreatic cancer and unfortunately promising preclinical results have not translated into a clinical response with the exception of MSI-H pancreatic cancer which constitutes only ~ 1% of the patients [8, 9]. To date, many theories have been proposed to explain disappointing outcomes with immunotherapy and targeted therapies while the microenvironment of pancreatic cancer has been deemed to be one of those major key factors for the failures.

**Fig. 1:**
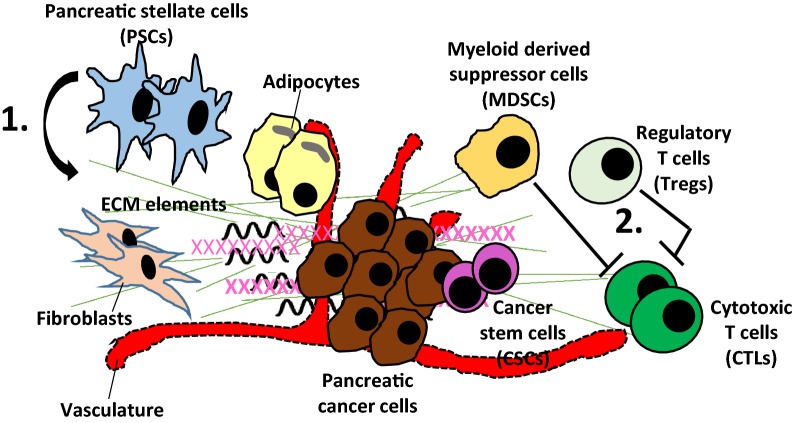
Unique characteristics of pancreatic cancer microenvironment. Pancreatic cancer stroma is enriched with pancreatic stellate cells (PSCs) that produce excessive amounts stromal elements such as collagens, laminin and fibronectin leading to desmoplasia, a process, which produces a hypovascular microenvironment, impairing local drug delivery, rendering tumors resistant to chemotherapeutics. Cancer stem cells (CSCs), which are known to be multidrug resistant also play a role in chemoresistance [1]. Pancreatic cancer microenvironment is “highly” infiltrated by a variety of immunosuppressive cell types such as myeloid derived suppressor cells (MDSCs) and regulatory T cells (Tregs) that mitigate the effector function of cytotoxic T cells (CTLs), leading to immune evasion. That constitutes an important factor for ineffectiveness of immunotherapies in pancreatic cancer along with the hypoimmunogenic nature of the cancer due to low mutation burden and lack of significant neoantigens [2]

The pancreatic cancer microenvironment harbors unique characteristics that directly impact the molecular behavior of cancer cells. It is known to be relatively dense and enriched by pancreatic stellate cells [10] which produce a redundant amount of stromal elements including collagens, laminin, and fibronectin; a process called desmoplasia [11]. Current evidence suggests that stellate cells are activated by proinflammatory cytokines orchestrated by myeloid-derived suppressor cells (MDSC) [12] which are frequently present in the pancreatic cancer stroma. Stellate cell-induced desmoplasia leads to a hypovascular microenvironment which configures the molecular signature of cancer cells. For example, microenvironment related hypoxia transforms cancer cell and induces modifications in the gene expression profile which facilitates adaptation to the continuously changing microenvironment [13]. Another study reported that hypoxia-inducible factor-1α (HIF-1α) upregulates the multidrug resistance gene, pointing to a distinct mechanism for stroma-induced chemotherapy resistance [14]. A mouse model of human pancreatic cancer cells showed increased expression of a variety of prosurvival genes such as cell cycle promoting genes (Cyclin B1), apoptosis inhibitor genes (Bcl-2 and survivin), and notably DNA repair genes (BRCA2 and Rad51) [15]. Moreover, upregulation of HIF-1α in cancer cells also reduces the efficacy of radiotherapy [16]. Taken together, current evidence indicates that pancreatic cancer microenvironment has direct and consequential effects on molecular characteristics of cancer cells by impacting the gene expression profile as well as altering drug delivery (Fig. 1).

In this article, we discuss potential impacts of the pancreatic cancer microenvironment on therapeutic approaches and targetability of this dense tumor stroma to optimize the efficacy of treatments for this aggressive disease.

## Targeting tumor microenvironment in combination with cytotoxic agents

**Fig. 2:**
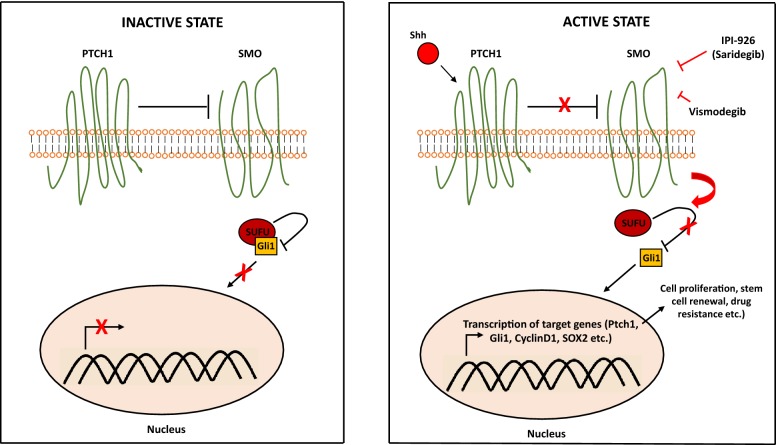
Sonic hedgehog signaling pathway. In the absence of sonic hedgehog ligand (Shh), the surface receptor Patched1 (PTCH1) inhibits Smoothened (SMO), resulting in sequestration of Gli1 in the cytosol by Suppressor of fused (SUFU). Binding of Shh to PTCH1 abolishes constitutive inhibition of SMO by PTCH1, leading to liberation of Gli1 from SUFU. Released Gli1, then, translocates to the nucleus, where it promotes gene expression. Sonic Hedgehog inhibitors IPI-926 (Saridegib) and Vismodegib block SMO activity

Chemotherapy resistance, one of the key causes for the aggressive nature of this disease was attributed to multiple factors related to tumor characteristics such as epithelial-to-mesenchymal transition (EMT) [17], increased cancer stem cells population [18] and hypovascular tumor microenvironment [19]. Stellate cell-induced desmoplasia was particularly intriguing as highly dense stromal tissue limits blood flow to cancer cells and consequently reduces the efficacy of drug delivery [20]. Studies suggest that sonic hedgehog signaling (Fig. 2) is the key driver of the desmoplasia process in pancreatic cancer [21]. This discovery was followed by a study where the investigators examined a sonic hedgehog inhibitor, IPI-926, in combination with gemcitabine and the authors reported transiently increased intratumoral concentration of gemcitabine in mouse models [22]. Consistent with these findings, another preclinical study investigating the ormeloxifene, a sonic hedgehog inhibitor, reported reduced desmoplasia leading to chemosensitization of pancreatic cancer cells to gemcitabine [23]. In a genetically engineered mouse model of pancreatic cancer, increased amounts of hyaluronan in the extracellular matrix was associated with poor vascular function, leading to impaired drug delivery; a phenomenon that was reversed by pegylated human recombinant PH20 hyaluronidase (PEGPH20) [24]. These promising preclinical findings led to the investigation of stroma depleting agents and sonic hedgehog inhibitors in clinical trials. PEGPH20 has been examined in combination with gemcitabine and nab-paclitaxel in a phase II study [25]. The authors reported better progression free survival in the investigational arm (HR 0.51; 95% CI 0.26–1.00; p = 0.048) and high levels of hyaluronan was found to be a predictor of response [25]. However, the combination of PEGPH20 with FOLFIRINOX was detrimental in the study by Ramanathan et al. [26] particularly due to increased toxicity leading to early termination of treatment in many patients enrolled in the investigational arm. The concerns created by the conflicting results presented by these studies will be addressed in the phase III trial of PEGPH20 in combination with gemcitabine and nab-paclitaxel [27]. A phase II study of IPI-926 (saridegib), a sonic hedgehog inhibitor, in combination with gemcitabine was conducted to enhance drug delivery but led to worse overall survival outcomes compared to the placebo arm [28]. In a Phase II clinical trial by Catenacci et al. [29] vismodegib did not improve overall response rate and no significant enhancement in the delivery of gemcitabine to the tumor microenvironment was noted. These disappointing results from clinical trials triggered “back to the bench” studies, in which stromal elements of the tumor microenvironment were indeed shown to restrain the pancreatic cancer cells and inhibition of this process did not reverse drug resistance rather unleashed cancer cells that are highly metastatic [30, 31]. It is important to note that, sonic hedgehog signaling is significantly more active in pancreatic cancer stem cells [32] which may be the driver of the desmoplastic process. Therefore, reversal of hypovascular stroma may simply unleash the strain of aggressive cancer clones, ultimately potentiating their metastatic capacity [33]. Even though dense tumor stroma may be reducing the penetrance of chemotherapeutic agents to cancer cells, there may be other important factors for chemoresistance in pancreatic cancer such as increased expression of DNA repair pathway genes and upregulation of anti-apoptotic proteins [34–37].

At this juncture, studies combining stroma modulating agents in combination with cytotoxic agents have not demonstrated significant activity to change current clinical practice. Phase III trial of PEGPH20 with gemcitabine and nab-paclitaxel will likely provide more understanding on targetability of pancreatic cancer stroma in combination with cytotoxic agents. Future studies focusing on characterization of desmoplasia and its cause-effect relationship with pancreatic cancer stem cells may shed further light on druggability of the pancreatic cancer microenvironment in combination with cytotoxic agents.

## Targeting tumor microenvironment in combination with other targeted therapies

Targeted therapy in pancreatic cancer has not substantially achieved progress particularly due to its complex signaling network and rebound activation of alternative growth signaling pathways [7]. Erlotinib, which is a small molecule tyrosine kinase inhibitor targeting epidermal growth factor receptor is currently the only FDA approved targeted therapy in combination with gemcitabine for metastatic pancreatic cancer albeit offers very limited therapeutic value [38]. There are only a limited number of studies in the literature that investigated the impact of pancreatic cancer stroma on targeted therapies. In a study by Lonardo et al. [39] inhibition of the Nodal/Activin pathway in pancreatic cancer stem cells abolished their self-renewal capacity and in vivo tumorigenicity, and abrogated the gemcitabine resistance of orthotopically inoculated stem cells. Yet, gemcitabine sensitivity was unsatisfactory in the engrafted human cancer tissue with stroma which was reversed by a sonic hedgehog pathway inhibitor [39]. In another preclinical study, a mammalian target of rapamycin (mTOR) inhibitor in combination with a sonic hedgehog inhibitor led to better disease control and chemosensitized the cancer stem cells to cytotoxic agents [40]. These studies support the notion that combining targeted therapies with stroma modifying agents may carry therapeutic significance if targeted agents have potential clinical activity on chemoresistant cancer cells, particularly cancer stem cells. It is important to note that although sonic hedgehog signaling directed therapies may generate a stroma depleting effect, they should not be considered as targeted therapies for cancer stem cells as pancreatic cancer stem cells do not appear to be addicted to this pathway [28, 29]. Moreover, even though these agents can be considered in strategies to enhance drug delivery to the tumor microenvironment, the literature is also not consistent on this effect [23, 29].

## Targeting tumor microenvironment in combination with immunotherapy

The efficacy of immunotherapy in pancreatic cancer has been widely studied in preclinical and clinical studies. However, at this time the benefit of immunotherapy appears to be limited to only microsatellite instability-high (MSI-H) pancreatic cancer patients who had an objective clinical response with pembrolizumab in a phase II clinical trial [41]. Single agent immune check point inhibitors and cancer vaccines have not improved clinical outcomes in microsatellite stable (MSS) pancreatic cancer patients [42, 43] and the benefit combined models yet to be determined [44] (NCT03190265, clinicaltrials.gov). Although the exact mechanisms of resistance to immunotherapies remain elusive, the current state-of-the-science suggests that pancreatic cancer is a hypoimmunogenic tumor due to low mutation burden and lack of significant neoantigens which are tumor-specific mutated peptides [45]. Moreover, stellate-cell induced desmoplastic reaction also creates a physical barrier for CD8+ T cells recruitment leading to a safe haven for the growth of cancer cells and evasion of immune response [46]. Beside these facts, the microenvironment of pancreatic cancer is also infiltrated by a variety of immunosuppressive inflammatory cells such as MDSC and regulatory T cells (Tregs) [3]. These cells are known to mitigate the effector function of cytotoxic T cells leading to immune evasion (Fig. 2), which points out that pancreatic cancer stroma could be an important factor for ineffective anti-cancer immune response. Interestingly, increased Tregs in the microenvironment also correlates with circulating Tregs [47] suggesting there may be synchronized systemic and microenvironmental immune dysregulation. Although preclinical studies suggested there may be an improvement in immune response upon depletion of Tregs [48], this benefit has not been confirmed in clinical studies [43]. In the ECLIPSE trial, depletion of Tregs in the tumor microenvironment with cyclophosphamide in combination with GVAX vaccine did not lead to a significant improvement in anti-cancer immune response with the clinical outcomes being consistently disappointing [49]. There is preliminary evidence that cancer vaccine in combination with low dose cyclophosphamide may optimize the tumor microenvironment and sensitize cancer cells to immune checkpoint inhibitors [50]. This triple combination approach is currently being investigated in clinical trials in different clinical settings (Table 1). MDSCs have been targeted in a mouse model and the authors reported that education of these cells may enhance the efficacy of immune checkpoint inhibitors in pancreatic cancer [51]. Carcinoma-associated fibroblasts have also been linked to downregulation of anti-cancer immune response [52]. A preclinical study with mouse models showed that targeting carcinoma-associated fibroblasts may enhance the efficacy of anti-PD-L1 based therapy [53]. However, another preclinical study reported that depletion of carcinoma-associated fibroblast may indeed trigger immunosuppuression with increased infiltration of Tregs which was reversed by an anti-CTLA4 antibody [54]. Inhibition of the sonic hedgehog pathway by use of nanoparticles in combination with cytotoxic agents was also reported to increase tumor vascularity without altering fibroblasts and stromal collagen, leading to an increase in T cell infiltration in the tumor microenvironment with improvement in mouse survival [55]. It is important to note that this approach without the use of nanoformulation has been investigated in clinical trials and did not show any substantial improvement in survival outcomes [29]. Currently, other approaches to modify the tumor microenvironment are also being investigated. A preclinical study suggested reduction of fibrosis in the tumor microenvironment via inhibition of focal adhesion kinase (FAK), enhancing T cell responsiveness to PD-1 based check point inhibitors [56]. Overall, the combination of stroma modulating agents with immunotherapy shows limited clinical benefit in pancreatic cancer and is to be further investigated by future studies. There is a hope that further studies may uncover potentials of immunotherapy in combination with stroma targeting agents.

**Table 1:** Selected ongoing clinical trials investigating immunotherapy based therapies targeting pancreatic cancer microenvironment

Identifier	Trial design/phase/current status	Rationale	Study group
NCT02243371	GVAX pancreas vaccine (with cyclophosphamide) and CRS-207 with or without nivolumab/phase2/active, not recruiting	Priming of tumor microenvironment and T cells/activation of effector immune system	Previously treated metastatic pancreatic adenocarcinoma
NCT03153410	Pilot study of cyclophosphamide, pembrolizumab, GVAX, and IMC-CS4 (LY3022855) in patients with borderline resectable adenocarcinoma of the pancreas/phase 1/recruiting	Depleting/inhibiting negative regulators of immune response and priming of tumor microenvironment	Borderline resectable adenocarcinoma
NCT03190265	Study of CRS-207, nivolumab and ipilimumab with or without GVAX pancreas vaccine (with CY) in patients with pancreatic cancer/recruiting	Priming of tumor microenvironment and T cells/activation of effector immune system	Previously treated metastatic pancreatic adenocarcinoma
NCT03161379	GVAS pancreas vaccine with cyclophosphamide in combination with nivolumab and SBRT for patients with borderline resectable pancreatic cancer/phase 2/recruiting	Priming of tumor microenvironment and T cells/activation of effector immune system	Borderline resectable pancreatic adenocarcinoma
NCT02451982	Neoadjuvant/adjuvant GVAX pancreas vaccine (with CY) With or without nivolumab and urelumab trial for surgically resectable pancreatic cancer/phase1-2/recruiting	Priming of tumor microenvironment and activation of effector immune cells	Surgically resectable pancreatic adenocarcinoma
NCT02648282	Study with cyclophosphamide pembrolizumab, GVAX, and SBRT in patients with locally advanced pancreatic cancer/phase2/recruiting	Priming of tumor microenvironment and activation of effector immune cells	Locally advance pancreatic adenocarcinoma

## Future perspectives

At this juncture, the pancreatic cancer microenvironment remains a dilemma for scientists particularly due to contradictory findings from reported preclinical and clinical studies. Current evidence fails to substantially support the notion of using stroma modifying agents to enhance cytotoxic drug delivery in pancreatic cancer. It is important to note that chemoresistance of pancreatic cancer is not solely related to the dense and fibrotic microenvironment and there are intrinsic factors that are associated with reduced sensitivity to cytotoxic agents as discussed above. Notably, phylogenesis of pancreatic cancer is not independent of its microenvironment and a high degree of hypoxia led by desmoplasia likely impacts the evolutionary expression profile throughout the development of cancer. Therefore, reversal of fibrotic stromal tissue without additive strategies targeting chemoresistant cancer cells will likely not be effective on the aggressive behavior of pancreatic cancer and may also lead to detrimental outcomes. However, targeted agents that are proven to be “highly” effective on multidrug resistant cancer cells may be an approach that can be investigated in preclinical studies and can be further examined in clinical trials if highly promising. Further studies on molecular biology for pancreatic cancer are also warranted to better understand the mechanisms leading to chemoresistance, which may trigger new approaches for targeted therapies in combination with stroma modulating agents.

The efficacy of immunotherapy in pancreatic cancer has been so far disappointing in MSS patients. The lack of clinical response may partially be explained by the microenvironment, which is predominantly infiltrated by immune suppressive cells such as Tregs and MDSCs. Strategies targeting stroma and immune regulatory cells have not yielded significant clinical response at least partially due to hypoimmunogenic nature of pancreatic cancer. Cancer vaccines which are designed to prime immune cells and optimize the tumor microenvironment have not led to significant clinical progress due to rebound activation of inhibitory signals on cytotoxic T cells such as PD-1. The additive role of immune checkpoint inhibitors to GVAX is currently being investigated which may lead to a measurable anti-cancer immune response. Stromal remodeling therapies may also enhance the generation of secondary and tertiary lymphoid structures and potentiate the anti-cancer effect if combined immunotherapy approaches achieve substantial clinical response.

## Conclusion

To date, the unique features of the pancreatic cancer microenvironment have been extensively studied. However, the complexity of interaction between the microenvironment and cancer cells remains to be better characterized. The failure of stroma modifying agents revealed that we have not yet comprehended exact consequences of this dual interaction on the development and progression of pancreatic cancer. Although the current state of the science regarding the complex microenvironment of pancreatic cancer has not revealed druggable targets, ongoing and future studies may shed more light to unknowns of this complex interaction and uncover more facts on this dilemma which may provide further therapeutic tools to scientists and physicians to advance treatment options for this malicious disease.

## Abbreviations

Tregs: regulatory T cells
MDSC: myeloid-derived suppressor cells
HIF-1 α: hypoxia-inducible factor-1α
EMT: epithelial-to-mesenchymal transition
PEGPH20: pegylated human recombinant PH20 hyaluronidase
PD-1: programmed cell death protein-1
CTLA4: cytotoxic T-lymphocyte associated protein 4
MSS: microsatellite stable
MSI-H: microsatellite instability-high
